# Brainstem lesions are associated with diffuse spinal cord involvement in early multiple sclerosis

**DOI:** 10.1186/s12883-022-02778-z

**Published:** 2022-07-19

**Authors:** Michaela Andelova, Karolina Vodehnalova, Jan Krasensky, Eliska Hardubejova, Tereza Hrnciarova, Barbora Srpova, Tomas Uher, Ingrid Menkyova, Dominika Stastna, Lucie Friedova, Jiri Motyl, Jana Lizrova Preiningerova, Eva Kubala Havrdova, Bénédicte Maréchal, Mário João Fartaria, Tobias Kober, Dana Horakova, Manuela Vaneckova

**Affiliations:** 1 Department of Neurology and Center of Clinical Neuroscience, First Faculty of Medicine, Charles University and General University Hospital, Katerinska 30, Praha 2, Prague, Czech Republic; 2 Department of Radiology, First Faculty of Medicine, Charles University and General University Hospital, Prague, Czech Republic; 3 2nd Department of Neurology, Faculty of Medicine, Comenius University, Bratislava, Slovakia; 4 Advanced Clinical Imaging Technology, Siemens Healthcare AG, Lausanne, Switzerland; 5 Department of Radiology, Lausanne University Hospital and University of Lausanne, Lausanne, Switzerland; 6 Signal Processing Laboratory (LTS 5), École Polytechnique Fédérale de Lausanne (EPFL), Lausanne, Switzerland

**Keywords:** Multiple sclerosis, Infratentorial lesions, Spinal cord, Focal and diffuse lesions, thalamus

## Abstract

**Background:**

Early infratentorial and focal spinal cord lesions on magnetic resonance imaging (MRI) are associated with a higher risk of long-term disability in patients with multiple sclerosis (MS). The role of diffuse spinal cord lesions remains less understood. The purpose of this study was to evaluate focal and especially diffuse spinal cord lesions in patients with early relapsing-remitting MS and their association with intracranial lesion topography, global and regional brain volume, and spinal cord volume.

**Methods:**

We investigated 58 MS patients with short disease duration (< 5 years) from a large academic MS center and 58 healthy controls matched for age and sex. Brain, spinal cord, and intracranial lesion volumes were compared among patients with- and without diffuse spinal cord lesions and controls. Binary logistic regression models were used to analyse the association between the volume and topology of intracranial lesions and the presence of focal and diffuse spinal cord lesions.

**Results:**

We found spinal cord involvement in 75% of the patients (43/58), including diffuse changes in 41.4% (24/58). Patients with diffuse spinal cord changes exhibited higher volumes of brainstem lesion volume (*p* = 0.008). The presence of at least one brainstem lesion was associated with a higher probability of the presence of diffuse spinal cord lesions (odds ratio 47.1; 95% confidence interval 6.9–321.6 *p* < 0.001) as opposed to focal spinal cord lesions (odds ratio 0.22; *p* = 0.320). Patients with diffuse spinal cord lesions had a lower thalamus volume compared to patients without diffuse spinal cord lesions (*p* = 0.007) or healthy controls (*p* = 0.002).

**Conclusions:**

Diffuse spinal cord lesions are associated with the presence of brainstem lesions and with a lower volume of the thalamus. This association was not found in patients with focal spinal cord lesions. If confirmed, thalamic atrophy in patients with diffuse lesions could increase our knowledge on the worse prognosis in patients with infratentorial and SC lesions.

**Supplementary Information:**

The online version contains supplementary material available at 10.1186/s12883-022-02778-z.

## Background

Multiple sclerosis (MS) is the most common chronic disabling autoimmune and neurodegenerative neurological disease in young adults characterized by accumulation of focal demyelinating lesions, widespread chronic neuroinflammation and neuronal loss in the brain and spinal cord (SC). The wide heterogeneity of clinical characteristics, genetics, pathogenesis, and response to treatments observed within MS population calls for reliable early prognostic markers to personalize treatment and improve the design and analysis of therapeutic trials and observational studies. Magnetic resonance imaging (MRI) of the brain and SC plays a crucial role both in the diagnosis and in the monitoring of disease activity. The clinical and prognostic relevance of infratentorial (brainstem and cerebellum) and SC lesions related to their strategic anatomical functional localization has been recognized early on [1, 2]. Subsequent studies have confirmed a higher risk of conversion to clinically definite MS, more aggressive disease, disability progression and developing secondary progressive disease associated with infratentorial and SC lesions [3–7]. Two different types of SC lesions that differ histopathologically by their degree of myelination, gliosis, and by the involvement of gray matter (GM) and white matter (WM) have been described in MS. Firstly, sharply demarcated focal lesions (FL) that are rather small, covering less than two SC segments and usually less than half of the cord area [8, 9], and secondly, diffuse areas of abnormal signal intensity lacking a well-demarcated border [10]. Okuda’s group defined diffuse SC lesions (DL) as areas or the coalescing appearance of multifocal lesions on sagittal view [11]. DL are associated with higher disability [12] and SC atrophy [13–15], however, they are not incorporated into current MS diagnostic criteria [16] as their assessment is considered to be not reliable and unspecific. SC atrophy is present predominantly in the cervical SC, occurs early in the disease course [17] and shows a significant correlation with the subsequent progression of physical disability [18, 19]. Therefore, the need arises to find and understand DL and other early biomarkers associated with future SC atrophy. The current study aimed to evaluate SC involvement in early MS and to assess the association of FL and DL with the volume and topography of intracranial lesions and the regional and global brain volumes and spinal cord volume. Our hypotheses were twofold: (i) patients with DL have a higher volume and/or a different distribution of intracranial white matter lesions than patients with FL or normal appearing SC and (ii) patients with DL have a lower SC volume and/or lower global and regional brain volumes than patients with only FL or normal appearing SC.

## Methods

### Subjects

We included 58 patients with early relapsing-remitting MS diagnosed according to McDonald criteria with a disease duration < 5 years. Patients with MS underwent their routine annual brain and SC MRI examination and a complete neurological examination including the EDSS (Expanded Disability Status Scale). From 102 healthy controls (HC) who were recruited as a control group for MS research, we selected 58 HC matched for sex and age. HC underwent brain and SC MRI on the same scanner using the same imaging protocol as patients with MS. The study was conducted in accordance with the Good Clinical Practice Guidelines. MRI and clinical examinations were performed as part of standard care and monitoring within our MS Center. The local Ethics Committee approved the examination of both MS patients and HC. Each participant involved in this study provided informed consent before individual data were collected, stored, and analysed. Demographic and clinical characteristics (EDSS and its functional system scores and initial symptoms) of the 58 patients and the HC matched for sex and age are shown in Supplementary Table 1.

### Magnetic resonance acquisition

All patients and HC were scanned at 3 T (MAGNETOM Skyra, Siemens Healthcare, Erlangen, Germany) using the same protocol. This included a 3D-T2W sequence in the transversal plane for SC volume measurement, and a sagittal T2WI-Fat-Sat and PDW sequences for the assessment of FL and DL. The SC was assessed between the cervicomedullary junction (C1 level) and the Th4 level. Intracranial lesions were segmented on a 3D fluid attenuated inversion recovery (FLAIR) sequence and magnetization-prepared rapid acquisition with gradient echo (MPRAGE) was used to assess global and regional brain volumes. The parameters of the sequences are summarized in Table 1.

**Table 1:** Details of the MRI protocol. MRI System: MAGNETOM Skyra; Siemens Healthcare, Erlangen, Germany

Sequence	Purpose		
T2W SPACE axial	Spinal cord volume measurement (ScanvView.cz)	Voxel size (mm3)	0,6 × 0,6 × 1
		TR/TE (ms)	1500/133
		Flip Angle(°)	150
		Field of view (mm)	200
T2WI-Fat-Sat sagittal	Spinal cord focal lesions and diffuse changes assessment	Voxel size (mm3)	0,6 × 0,6 × 1
		TR/TE (ms)	2800/84
		Flip Angle(°)	160
		Field of view (mm)	220
PDWI sagittal	Spinal cord focal lesions and diffuse changes assessment	Voxel size (mm3)	0,7 × 0,7 × 2
		TR/TE (ms)	2500/7.6
		Flip Angle(°)	160
		Field of view (mm)	220
3D FLAIR	Intracranial lesion segmentation (LeMan-PV Prototype)	Voxel size (mm3)	1x1x1
		TR/TE/TI (ms)	5000/397/1800
		Flip Angle(°)	120
		Field of view (mm)	256
3D MPRAGE	Global and regional brain volumes segmentation (Morphobox Prototype)	Voxel size (mm3)	1x1x1
		TR/TE/TI (ms)	2300/2.26/900
		Flip Angle(°)	9
		Field of view (mm)	256

### Spinal cord volume measurement and detection of spinal cord involvement

**Fig. 1:**
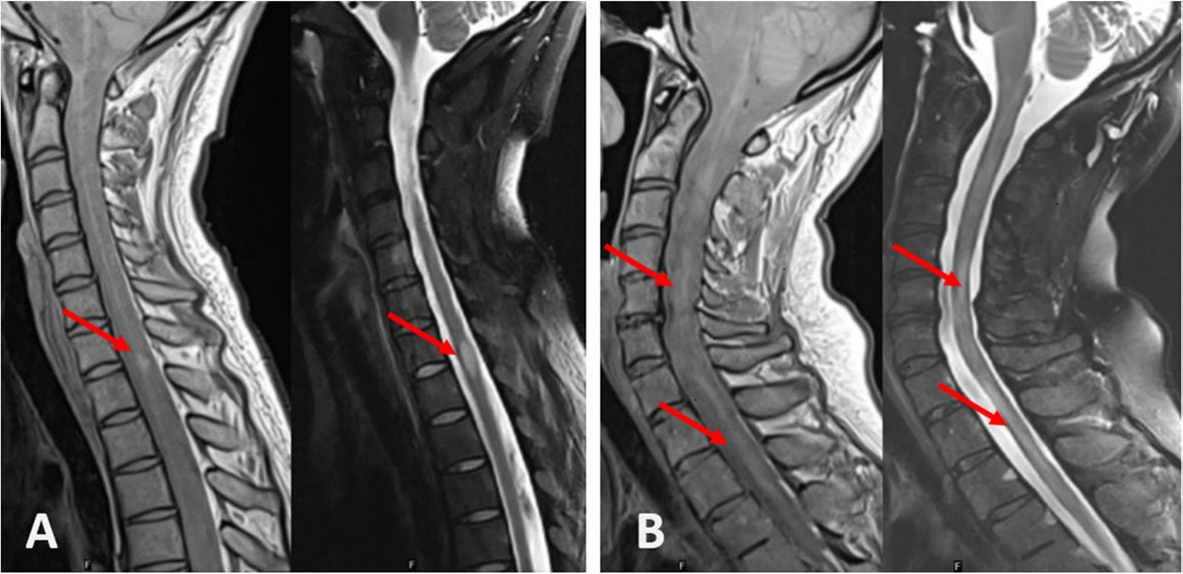
Examples of focal and diffuse spinal cord lesions (left PD sequence and right T2W sequence) on diagnostic MRI of the cervical and upper thoracal spinal cord in two patients with MS. **A**: focal lesion without diffuse lesions in a 24-year-old patient (EDSS 2.0) **B**: diffusely abnormal signal without clearly demarcated focal lesions in a 40-year-old patient (EDSS 3.5)

The volume of SC was quantified by measuring the MUCCA (mean upper cervical cord cross-sectional area) by using a semi-automatic edge finding tool implemented in ScanView.cz [20], as described previously [15]. Sagittal T2W fat-sat and proton density (PDW) sequences were used to assess the number and localisation of FL and/or DL. The classification of SC abnormalities was based on the work of Lycklama á Nijeholt et al. [21, 22]. An experienced neuroradiologist with over 20 years of experience in MS imaging (M.V.) and a neurologist (M.A.) manually assessed the absence or presence of FL and/or DL. FL were defined as sharply demarcated T2/PDW-hyperintense areas and DL were defined as homogenously increased signal in multiple SC segments in the T2/PDW sequence. Subsequently, the patients were classified into four groups (SC ‘phenotypes’): 1. Patients with normal appearing SC; 2. Patients with only FL; 3. Patients with both FL and DL and 4. Patients with only DL. In case of disagreement, the case was reviewed and a decision was reached by consensus. As DL can be ambiguous, two additional neurologists with experience in MS (D.H. and D.S.) independently assessed SC lesions in all patients to assess inter-rater agreement. Examples of focal and diffuse lesions are shown in Fig. 1.

### Volume-based morphometry of the brain and segmentation of the intracranial lesions

Regional brain volume calculations were estimated by using MorphoBox prototype software (Siemens Healthcare, Erlangen, Germany) [23]. MorphoBox splits the segmentation of anatomical structures into two sequential steps: 1) classification of total intracranial volume (TIV) voxels in brain tissue - cerebrospinal fluid (CSF), GM, WM, and 2) brain structure segmentation by combining tissue probability maps obtained in the previous step with anatomical masks derived from a single-subject template via non-rigid registration. Global and regional brain volumes were normalized using the TIV (proportional method). Intracranial lesion volumes were estimated on FLAIR images using an automated and quantitative MS lesion segmentation method (LeManPV prototype software, Siemens Healthcare, Erlangen, Germany), which has been previously developed and tested in patients in early stages of MS disease and low disability and allows for a classification of lesions based on their topography, including also brainstem and cerebellar lesions [24]. The lesion masks were visually inspected by a neurologist (M.A.) and when necessary, manually corrected.

### Statistical analysis

SPSS software (Version 20, Chicago, IL, USA) was used for case-control matching and statistical analyzes. Case-control matching was used to create pairs of sex and age-matched HC and patients (tolerance for age difference was established at 2 years). The inter-rater reliability was calculated using Cohen’s kappa. To assess the normality of the distributions, we used visual inspection of histograms and Q-Q plots and the Shapiro–Wilk test. Due to the limited number of patients in the four SC phenotypes mentioned above and due to the absence of differences between the groups with respect to age, disease duration, or EDSS, the patients were dichotomized according to the presence/absence of DL for statistical comparisons. Intracranial lesion volumes between patients with and without DL were compared using the Mann-Whitney U test. Differences in global and regional brain volumes and SC volumes between HC, patients with and without DL, were evaluated using the One-way ANOVA and Kruskal-Wallis H test. Post hoc pairwise comparisons were made using Tukey’s method in the case of ANOVA and Bonferroni correction in the case of the Kruskal-Wallis H test. The corrected significance values are reported. We employed two separate binary logistic regression analyses to explore predictive characteristics of the presence of DL and FL, respectively. Independent variables were sex, age, disease duration, total volume of intracranial lesions, and the presence of brainstem and/or cerebellar lesions.

## Results

### Reliability of the assessment of SC abnormalities

We found a substantial agreement among raters for normal appearing SC (Cohen’s kappa κ = 0.79), isolated FL (κ = 0.71), for a combination of FL and DL (κ = 0.67) and for DL in the absence of FL (κ = 0.84).

### SC involvement in patients with early MS

Fifteen patients (25.9%) had normal appearing SC. SC abnormalities were found in 43 of 58 (74.1%) patients with early MS. 19 (32.8%) patients had only focal SC lesions. 24 (41.4%) demonstrated DL, 14 (24.1%) of which had both FL and DL, and 10 (17.2%) had only DL.

### Intracranial lesion volumes and topography in patients with and without diffuse SC abnormalities

**Fig. 2:**
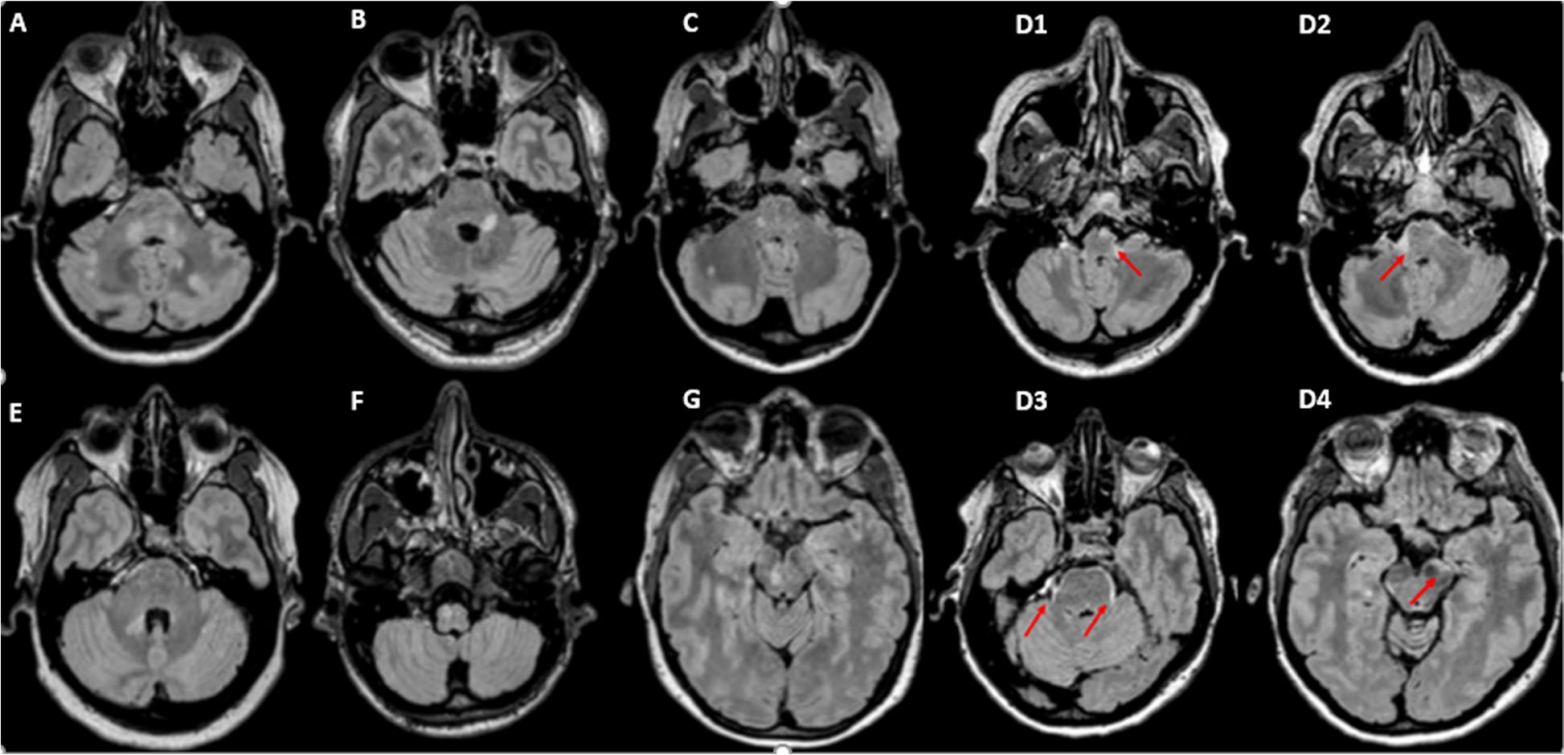
Representative images of brainstem and cerebellar lesions in patients with early MS. Typical lesions in cerebellar peduncles and discrete subpial ‘linings’ along the periphery of the brainstem (marked with arrows). **A**: 40-year-old patient, DD 4 months, EDSS 3.5; **B**: 44-year-old patient, DD 1 month, EDSS 2.0; **C**: 31-year-old patient, DD 25 months, EDSS 1.5; **D1-D4**: 51-year-old patient, DD 4 months, EDSS 4.0; **E**: 45-year-old patient, DD 3 months, EDSS 1.0; **F**: 23-year-old patient, DD 1 month, EDSS 2.0; **G**: 40-year-old patient, DD 4 months, EDSS 2.0. DD = disease duration, EDSS = Expanded Disability Status Scale

**Fig. 3:**
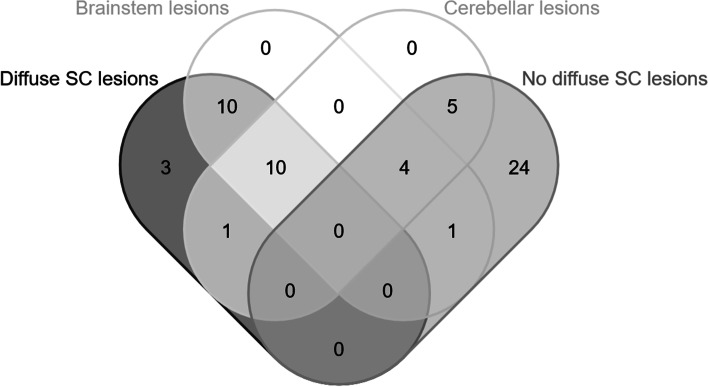
Association between spinal cord involvement and the presence of brainstem and cerebellar lesions

The total volume of intracranial lesions and the volumes of the lesions in the frontal, temporal, parietal, and occipital lobes did not differ between patients with DL and without DL. Patients with DL had a larger volume of brainstem lesions (*p* = 0.008) than patients without DL and the volume of cerebellar lesions was borderline significant (*p* = 0.05 after correction for false discovery rate) (Table 2). Infratentorial lesions were detected in 20 of 24 (83.3%) patients with DL and 10 of 34 (29.4%) patients without DL. Representative images of brainstem and cerebellar lesions are shown in Fig. 2. The different types of SC involvement and their association with the presence of infratentorial lesions are shown in Fig. 3.

**Table 2:** Intracranial lesion volume in particular regions in patients with and without diffuse spinal cord lesions

	Patients without DL	Patients with DL	Statistical comparison (Mann-Whitney U-test)	
n (%)	34 (29.4)	24 (41.3)	*p*-value	*p*-value after FDR correction
Total intracranial lesion volume (cm^3^)	6.0 ± 8.1	10.1 ± 11.0	0.024	0.064
Frontal lesion volume (cm^3^)	1.1 ± 1.7	2.0 ± 2.8	0.165	0.165
Temporal lesion volume (cm^3^)	0.5 ± 1.1	1.1 ± 1.6	0.122	0.14
Parietal lesion volume (cm^3^)	1.1 ± 2.0	2.3 ± 3.6	0.065	0.104
Occipital lesion volume (cm^3^)	0.4 ± 0.4	0.7 ± 0.8	0.067	0.09
Deep hemispheric lesion volume (cm^3^)	2.8 ± 3.6	3.8 ± 2.7	.018	0.072
Cerebellar lesion volume (cm^3^)	0.01 ± 0.05	0.05 ± 0.13	0.025	**0.05**
Brainstem lesion volume (cm^3^)	0.01 ± 0.03	0.1 ± 0.15	0.001	**0.008**
n with- / n without infratentorial lesions	17 / 17	20 / 4		

*DL* Diffuse lesions, *FDR* False discovery rate

### Predicting the occurrence of diffuse and focal SC lesions

In binary logistic regression analysis with the presence of DL as a dependent variable and demographic and intracranial lesions as independent variables, only the presence of brainstem lesions was associated with a higher probability of DL (OR 47.1; 95% CI 6.9–321.61; *p* < 0.001). The volume of supratentorial lesions and the presence of cerebellar lesions were not associated with DL. The proportion of explained variance in DL expressed by Nagelkerke’s R was 55% (Table 3). In the second analysis with the presence of FL as a dependent variable and with the same independent variables, neither intracranial lesion volume nor brainstem or cerebellar lesions were associated with the presence of FL (OR 0.22; 95% CI 0.01–4.3; *p* = 0.32) (Supplementary Table 2).

**Table 3:** Hierarchical binary logistic regression analysis investigating the relative contributions of covariates in predicting the presence of diffuse SC abnormalities

	Model 1			Model 2			Model 3		
	OR	95% CI	p	OR	95% CI	p	OR	95% CI	p
Sex (female = reference)	1.76	0.51–6.11	.372	2.05	0.57–7.41	.271	2.12	0.42–10.79	.276
Age	0.96	0.89–1.04	.333	0.96	0.88–1.04	.276	.94	0.85–1.04	.284
Disease duration	1.05	0.67–1.64	.835	1.02	0.65–1.6	.938	1.43	0.78–2.63	.153
Intracranial lesion volume				1.06	0.99–1.13	.103	1.02	0.94–1.11	.606
Presence of brainstem lesions							**47.10**	**6.9–321.61**	**<.001**
Presence of cerebellar lesions							0.29	0.04–2.47	.259
Hosmer and Lemeshow chi2/p	3.07 / 0.93			7.81 / 0.45			3.54 / 0.9		
Chi2 /Signif. of the step (model)	1.69 /0.69 (0.64)			4.95 / 0.071 (0.29)			35.01 / 0.001 (0.001)		
Nagelkerke R^2^ for the model	0.04			0.11			0.55		

Dependent variable: presence of diffuse spinal cord abnormalities. *CI* Confidence interval, *OR* Odds ratio; *p* = level of significance. Hosmer–Lemeshow statistics indicate a poor fit if the significance value is less than 0.05

### Spinal cord and brain volume in patients with early MS with and without DL

**Fig. 4:**
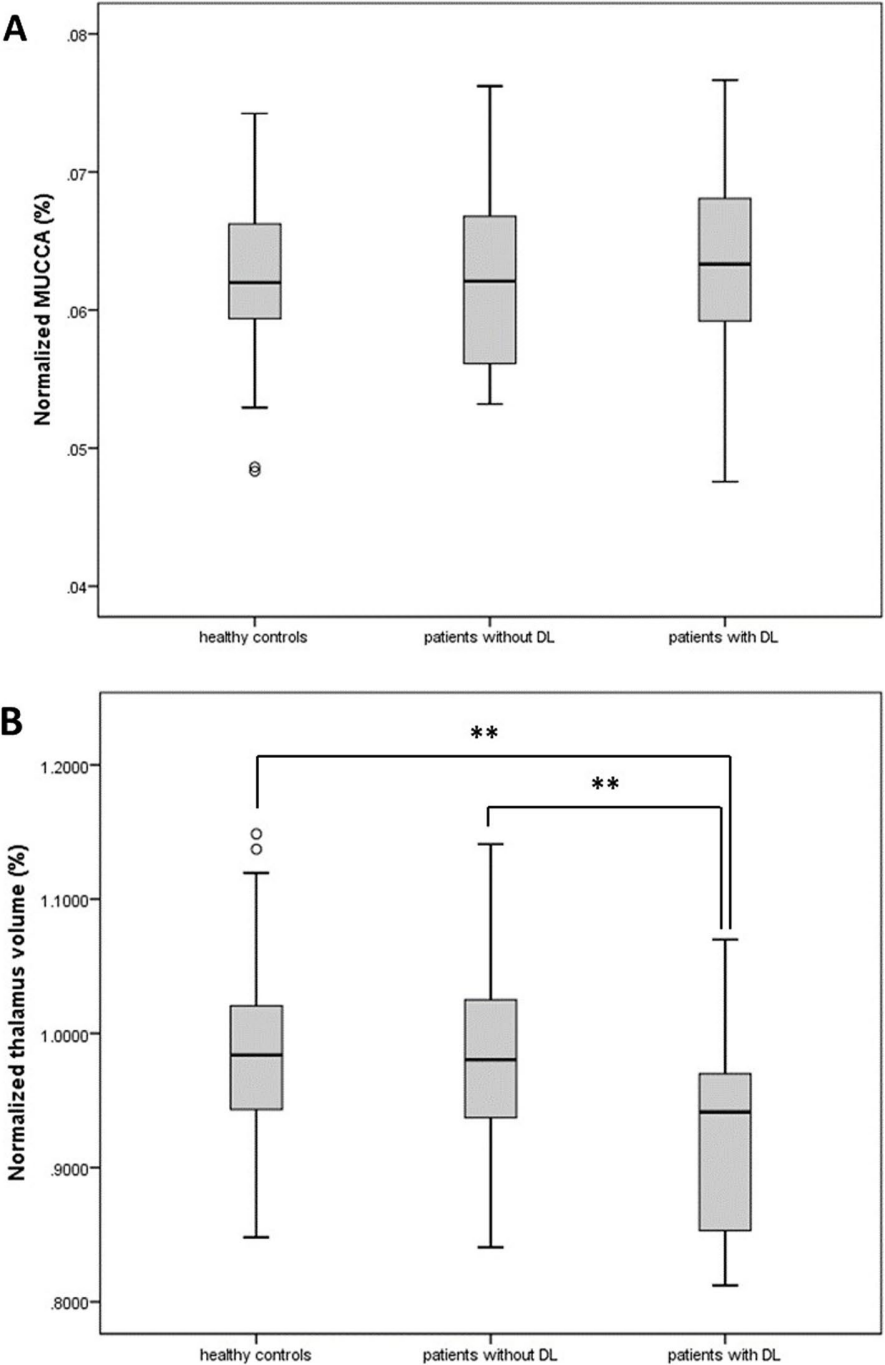
Comparison of (**A**) normalized MUCCA and (**B**) normalized thalamus volume among healthy controls, patients with and without diffuse spinal cord lesions. ** = *p* < 0.01. Legend: DL = diffuse lesions; MUCCA = mean upper cervical cord cross-sectional area

We have not found any differences in the absolute and normalized SC volume expressed as MUCCA (Fig. 4 A) and brain volume between HC and patients with and without DL. Among intracranial volumes, the thalamus was the only regional structure with significantly lower volume in patients with DL compared to patients without DL (*p* = 0.007) and HC (*p* = 0.002) (Fig. 4 B and Table 4).

**Table 4:** Pairwise comparisons between healthy controls, patients with and without DL (with post hoc corrections)

	Healthy controls (1)	Patients without DL (2)	Patients with DL (3)	Statistical comparison Mean difference (corresponding *p*-value)			Post-hoc pairwise comparison
n	58	34	24	1 vs. 2	1 vs. 3	2 vs. 3	
MUCCA (mm^2^)	89.3 ± 7.9	88.8 ± 7.62	88.5 ± 11.6	0.4 (.974)	0.8 (0.932)	0.3 (0.988)	1
MUCCA (%)	0.0624 ± 0.006	0.0623 ± 0.007	0.0627 ± 0.008	0.00009 (0.998)	− 0.00033 (0.997)	−0.00042 (0.968)	1
Brain volume (%)	80.3 ± 2.0	79.0 ± 2.9	79.3 ± 3.3	1.3 (0.062)	1.0 (0.272)	− 0.3 (0.897)	2
GM (%)	49.2 ± 2.1	48.6 ± 1.9	48.1 ± 2.4	0.6 (0.383)	1.0 (0.103)	0.4 (0.705)	1
WM (%)	31.2 ± 1.6	3.5 ± 1.9	31.2 ± 3.1	0.7 (0.283)	− 0.1 (0.989)	− 0.8 (0.362)	1
Lat. ventricles (%)	1.13 ± 0.58	1.35 ± .52	1.31 ± 0.77	− 0.2 (0.228)	− 0.2 (0.426)	0.03 (.978)	2
Brainstem (%)	2.2 ± 0.1	2.2 ± 0.1	2.2 ± 0.2	0.02 (0.823)	0.01 (0.923)	−0.001 (0.99)	1
Cerebellum (%)	8.8 ± 0.7	9.0 ± 0.5	8.8 ± 0.6	− 0.2 (0.497)	− 0.01 (0.998)	0.14 (0.666)	1
Corpus callosum (%)	4.1 ± 0.5	3.9 ± 0.4	3.9 ± 0.6	0.2 (0.19)	0.2 (0.367)	−0.02 (.981)	1
Thalami (%)	1.0 ± 0.1	1.0 ± 0.1	0.9 ± 0.2	0.002 (0.980)	**0.06 (0.002)**	**0.05 (0.007)**	1

All structures are volumes except MUCCA and corpus callosum, which are mean cross-sectional areas. The data for the structures are shown as volumes normalized to total intracranial volume, except MUCCA, which is presented as both raw area and normalized by TIV. All data are reported as means ± standard deviations. 1 = ANOVA with Tukey post hoc correction; 2 = Kruskal-Wallis test with Bonferroni post hoc correction; DL = diffuse lesions; MUCCA = mean upper cervical cord cross-sectional area

## Discussion

Using conventional MR imaging from routine clinical practice, we observed the involvement of SC in 75% of patients with an unexpectedly high occurrence of DL (41.4%) in a cohort of patients with relapsing-remitting MS with very short disease duration. The proportion of DL in our cohort was higher than reported in the work of Bot et al. who found DL in only 13% of very early MS [25], Lukas et al. who described DL in only 14.9% of patients (11.9% in RRMS, 17.4% in secondary progressive MS (SPMS) and 21.6% in primary progressive MS (PPMS)) [14] and Hua et al. who reported DL in 14.3% in the cervical cord and 19.1 in the thoracic cord of MS patients [11]. On the other hand, our findings are similar to those reported in a study by Coret et al. who found DL in 12 out of 32 (38%) early MS patients. However, the last-mentioned study included only patients with early MS and SC symptoms at onset, possibly resulting in an overestimation of the involvement of SC compared to the general population of MS. We perform diagnostic SC imaging in all patients with suspected MS regardless of the type of initial clinical presentation and the proportion of particular types of initial presentation in our cohort correspond to literature [26]. Therefore, we believe that the findings are representative for an unselected early MS population. Furthermore, the use of 3 T scanners might increase the sensitivity for DL compared to 1.5 T MRI used in previous studies [14, 25]. Finally, genetic factors such as the carriage of the human leukocyte antigen variant HLA- DRB1*1501 which could be associated with a higher prevalence of DL [27] might differ among the published cohorts.

We have not confirmed the hypothesis that patients with DL have a higher total intracranial lesion load than patients without DL. However, we have observed a larger volume of brainstem lesions in patients with DL than in patients without DL.  The presence of at least one brainstem lesion was associated with a substantially higher probability of DL, but did not increase the risk of FL. These findings might indicate an association of brainstem lesions with more severe SC involvement. Although the cross-sectional study does not allow for the assessment of the possible causal relationship between brainstem lesions and DL, there are several conceivable mechanisms by which brainstem and spinal cord involvement may be related. First, brainstem lesions might have an impact on tissue integrity not only at the site of the lesion, but also throughout the affected tract, thus connecting focal inflammatory activity and neurodegeneration in a more distant network, suggesting that axonal damage due to Wallerian degeneration (in supratentorial regions) plays a role, as was shown by Droby et al. [28]. However, previous work on the histopathological substrate of DL showed that they are mainly determined by demyelination and do not reflect axonal damage well [29–31]. Secondly, there could be a common pathophysiological process with predilection for infratentorial involvement that might be either genetically predetermined process or due to targeting antigens predominantly located infratentorially and in the SC. In fact, HLA-*DRB1* alleles may play a role in determining the severity and extent of SC damage in MS. The association of the carriership of HLA-DRB1*1501 with the extent of FL, the presence of DL, and greater disability was reported by two independent studies [27, 32]. An increase in T cell immunoreactivity against specific myelin antigens and the development of lesions in the brainstem and cerebellum was found in patients with MS and psoriasis who demonstrated a high frequency of carriage of the HLA-DRB1*1501 and HLA-DRB1*07 alleles [33, 34]. To explore the susceptibility to infratentorial and SC involvement further, it will be necessary to combine genotyping, immunology, and nonconventional MRI studies.

Our second hypothesis, that the presence of DL would be associated with a lower SC volume, was also not confirmed. Previous studies reported an association between DL and SC atrophy [13, 15], however patients in these studies had a longer disease duration and a higher degree of disability. Although SC atrophy is a well-established finding in progressive forms of MS, studies on clinically isolated syndrome (CIS) and relapsing-remitting MS (RRMS) have yielded inconsistent results [35]. The MUCCA may not reveal the presence of atrophy due to a combination of multiple pathological features, such as edema and gliosis, which can increase tissue volume, thereby counterbalancing the effects of demyelination and axonal loss [36]. Another potential explanation of the preserved SC volume could be that in early MS, there could be a ‘spinal cord reserve’ compensating for pathological-inclusive DL before SC atrophy develops. It will be necessary to investigate whether the presence of DL is a factor associated with (faster) spinal cord atrophy in MS in longitudinal studies.

Similarly to SC volume, we did not observe differences between patients with and without DL and HC in global and regional brain volumes, with the exception of thalami, which were smaller in patients with DL. Previous studies reported isolated thalamic atrophy in radiologically and clinically isolated syndromes and in early MS [37–39]. As mentioned above, damage to the SC tracts and brainstem might be associated with accelerated atrophy of distant brain structures [28]. Disconnection in the tracts that project from and to the thalamus could link more severe SC damage and loss of thalamic volume. In fact, in an experimental autoimmune encephalitis (EAE) model, it was demonstrated that inflammation of the spinothalamic tract at the SC level was associated with neuronal loss in the sensory ventral posterolateral nucleus of the thalamus [40]. A recent interesting study on MS phenotypes based on atrophy patterns described a small subgroup of patients characterized by prominent loss of thalamic and SC volume [41] that was associated with higher baseline MS severity scores, faster disability progression, and higher mean neurofilament levels, suggesting more prominent neuroaxonal damage and aggressive disease course. As thalamic atrophy is itself associated with accrual of disability [42, 43], its association with DL could increase knowledge of the worse prognosis in patients with infratentorial and SC lesions, if confirmed in a larger study.

Several limitations of the present study should be mentioned. First, despite the good agreement in DL assessment between the raters, the nonquantitative MRI may not be sufficiently sensitive to detect all pathological changes of the cord, and quantitative MRI measures such as diffusion tensor imaging (DTI), magnetization transfer ratio (MTR), T1 relaxometry, or MR spectroscopy would be needed to provide quantitative information beyond that that can be derived from macroscopically visible focal SC lesions and elucidate the microstructural nature of DL [44, 45]. Besides neuroimaging, the difference between the severity of FL and DL could be evaluated and objectively assessed by applying electrophysiological methods such as evoked potentials [46]. On the other hand, the consensus reading corresponds to the assessment in the clinical setting. Despite no clear guidelines for DL detection, we believe that DL indicates more severe pathology than focal lesions, that is, analogous to brain confluent lesions. Second, our assessment of FL was limited to counting lesion, which does not take into account neither the volume of affected tissue nor the location of the lesion that have been shown to be associated with disability [47]. Another caveat of the study is the limited coverage of the lower thoracic and lumbar spinal cord. Previous studies have shown a strong association between the presence of cervical cord- and thoracic cord damage [11], nevertheless, it is conceivable that we have underestimated the number of FL. Lastly, the cross-sectional nature of this study does not allow us to draw conclusions about the sequence and causality of the presence of brainstem and SC lesions and thalamic atrophy.

## Conclusions

The association of the diffuse, but not focal spinal cord lesions with the presence of brainstem lesions and with a lower volume of the thalamus might indicate different underlying pathology and/or severity of these two types of spinal cord involvement in MS. If confirmed, thalamic atrophy in patients with diffuse lesions could add to the knowledge of the worse prognosis in patients with infratentorial and SC lesions. Our findings further underscore the need for quantitative MRI markers to better understand different types of spinal cord involvement and their different prognostic value in MS.

## Supplementary Information

**Additional file 1: Supplementary Table 1.** Demographic and clinical characteristics of patients and healthy controls. EDSS = Expanded Disability Status Scale; FS = functional system; Age and disease duration are reported as means ± standard deviations. EDSS is reported as median (minimum – maximum). **Supplementary Table 2.** Hierarchical binary logistic regression analysis investigating the relative contributions of covariates in predicting the presence of focal spinal cord lesions. Legend: CI = confidence interval; OR: odds ratio, *p* = level of significance. Hosmer–Lemeshow statistics indicate a poor fit if the significance value is less than 0.05.

## Data Availability

The datasets used and/or analysed during the current study are available from the corresponding author on reasonable request.

## Abbreviations

CI: Confidence interval
CIS: Clinically isolated syndrome
CSF: Cerebrospinal fluid
DL: Diffuse (spinal cord) lesions
DTI: Diffusion tensor imaging
EAE: Experimental autoimmune encephalitis
EDSS: Expanded Disability Status Scale
FL: Focal (spinal cord) lesions
FS: Functional system
GM: Gray matter
HC: Healthy controls
HLA: Human leukocyte antigen
MPRAGE: Magnetization-prepared rapid acquisition with gradient echo
MS: Multiple sclerosis
MRI: Magnetic resonance imaging
MTR: Magnetization transfer ratio
MUCCA: Mean upper cervical cord cross-sectional area
OR: Odds ratio
PD: Proton density
PDW: Proton density weighted
PPMS: Primary progressive MS
RRMS: Relapsing-remitting MS
SC: Spinal cord
SPMS: Secondary progressive MS
WM: White matter
