# Supplementary material for: Brainstem lesions are associated with diffuse spinal cord involvement in early multiple sclerosis
Source: BMC Neurol. 2022 Jul 19;22:270. doi: 10.1186/s12883-022-02778-z (PMC9297663; doi:10.1186/s12883-022-02778-z)
Supplement: Supplementary file 1 — Additional file 1: Supplementary Table 1. Demographic and clinical characteristics of patients and healthy controls. EDSS = Expanded Disability Status Scale; FS = functional system; Age and disease duration are reported as means ± standard deviations. EDSS is reported as median (minimum – maximum). Supplementary Table 2. Hierarchical binary logistic regression analysis investigating the relative contributions of covariates in predicting the presence of focal spinal cord lesions. Legend: CI = confidence interval; OR: odds ratio, p = level of significance. Hosmer–Lemeshow statistics indicate a poor fit if the significance value is less than 0.05. [file 12883_2022_2778_MOESM1_ESM.docx]

|  | Healthy controls | Multiple sclerosis patients | | |
| --- | --- | --- | --- | --- |
|  |  | all | without diffuse SC changes | with diffuse SC changes |
| n (female/male) | 58 (44/14) | 58 (44/14) | 34 (27/7) | 24 (17/7) |
| Age (years) | 32.8 ± 7.5 | 32.8±7.4 | 31.7 ± 7.7 | 33.5 ± 7.2 |
| Disease duration(years) | - | 0.89±1.20 | 0.84 ± 1.08 | 0.96 ± 1.36 |
| EDSS | - | 2.0 (0-4.0) | 1.5 (0-4.0) | 2.0(1.0-4.0) |
| Visual FS score | - | 0 (0-6) | 0 (0-6) | 0 (0-2) |
| Pyramidal FS score * | - | 1 (0-3) | 1 (0-3) | 2 (1-3) |
| Brainstem FS score * | - | 0 (0-2) | 0 (0-1) | 0 (0-2) |
| Cerebellar FS score * | - | 0 (0-2) | 0 (0-1) | 0 (0-2) |
| Sensory FS score | - | 1 (0-3) | 1 (0-2) | 1 (0-3) |
| Initial symptoms | | | | |
| Optic neuritis n (%) | - | 19 (32.8) | 13 (38.2) | 6 (25) |
| Brainstem n (%) | - | 6 (10.3) | 3 (8.8) | 3 (12.5) |
| Spinal cord n (%) | - | 28 (48.3) | 16 (47.1) | 12 (50) |
| Hemispheric or polyregional n (%) | - | 5 (8.6) | 2 (5.9) | 3 (12.5) |

**Supplementary Table 1.** Demographic and clinical characteristics of patients and healthy controls

EDSS = Expanded Disability Status Scale; FS = functional system; Age and disease duration are reported as means ± standard deviations. EDSS is reported as median (minimum – maximum).

|  | Model 1 | | | Model 2 | | | Model 3 | | |
| --- | --- | --- | --- | --- | --- | --- | --- | --- | --- |
|  | **OR** | **95% CI** | **p** | **OR** | **95% CI** | **p** | **OR** | **95% CI** | **p** |
| Sex (female=reference) | 1.19 | .187-7.615 | .851 | .74 | .101-5.047 | .737 | .55 | .059-5.152 | .602 |
| Age | .94 | .854-1.042 | .252 | .93 | .839-1.039 | .205 | .93 | .825-1.042 | .205 |
| Disease duration | .95 | .475-1.932 | .905 | 1.07 | .512-2.223 | .863 | 1.23 | .533-2.819 | .633 |
| Intracranial lesion volume |  | | | .83 | .677-1.020 | .076 | .73 | .541- .989 | .042 |
| Presence of brainstem lesions |  |  |  |  | | | .22 | .012-4.299 | .320 |
| Presence of cerebellar lesions |  |  |  |  |  |  | 20.97 | .681 - 645.127 | .082 |
| Hosmer and Lemeshow chi2/p | 8.479 / .388 | | | 14.731 / .065 | | | 12.516 / .130 | | |
| Chi2 / Signif. of the step (model) | 1.374 / .712 (.712) | | | 6.545 / .023 (.162) | | | 11.198 / .098 (0.082) | | |
| Nagelkerke R^2^ for the model | .053 | | | .253 | | | 0.376 | | |

**Supplementary Table 2**. Hierarchical binary logistic regression analysis investigating the relative contributions of covariates in predicting the presence of focal spinal cord lesions.

Legend: CI = confidence interval; OR: odds ratio, p = level of significance. Hosmer–Lemeshow statistics indicate a poor fit if the significance value is less than 0.05
